# Brain phosphorylation of MeCP2 at serine 164 is developmentally regulated and globally alters its chromatin association

**DOI:** 10.1038/srep28295

**Published:** 2016-06-21

**Authors:** Gilda Stefanelli, Anna Gandaglia, Mario Costa, Manjinder S. Cheema, Daniele Di Marino, Isabella Barbiero, Charlotte Kilstrup-Nielsen, Juan Ausió, Nicoletta Landsberger

**Affiliations:** 1 San Raffaele Rett Research Unit, San Raffaele Scientific Institute, Milan, Italy; 2 Institute of Neuroscience, National Research Council (CNR), Scuola Normale Superiore Pisa, Italy; 3 Department of Biochemistry and Microbiology, University of Victoria, Victoria (BC), Canada; 4 Department of Informatics, Institute of Computational Science, Università della Svizzera Italiana, Lugano, Switzerland; 5 Department of Biotechnology and Life Sciences, University of Insubria, Busto Arsizio (VA), Italy; 6 Department of Medical Biotechnology and Translational Medicine, University of Milan, Milan, Italy

## Abstract

MeCP2 is a transcriptional regulator whose functional alterations are responsible for several autism spectrum and mental disorders. Post-translational modifications (PTMs), and particularly differential phosphorylation, modulate MeCP2 function in response to diverse stimuli. Understanding the detailed role of MeCP2 phosphorylation is thus instrumental to ascertain how MeCP2 integrates the environmental signals and directs its adaptive transcriptional responses. The evolutionarily conserved serine 164 (S164) was found phosphorylated in rodent brain but its functional role has remained uncharacterized. We show here that phosphorylation of S164 in brain is dynamically regulated during neuronal maturation. S164 phosphorylation highly impairs MeCP2 binding to DNA *in vitro* and largely affects its nucleosome binding and chromatin affinity *in vivo*. Strikingly, the chromatin-binding properties of the global MeCP2 appear also extensively altered during the course of brain maturation. Functional assays reveal that proper temporal regulation of S164 phosphorylation controls the ability of MeCP2 to regulate neuronal morphology. Altogether, our results support the hypothesis of a complex PTM-mediated functional regulation of MeCP2 potentially involving a still poorly characterized epigenetic code. Furthermore, they demonstrate the relevance of the Intervening Domain of MeCP2 for binding to DNA.

Epigenetics plays a fundamental role in the development and function of the mammalian nervous system. By linking DNA methylation to chromatin structure, the methyl-binding protein MeCP2 appears as a key player in reading the information encoded by methylated cytosines. Accordingly, the X linked *MECP2* gene is associated with several neurological disorders, of which Rett syndrome represents the most studied^1^.

MeCP2 was originally isolated as a ubiquitously expressed protein containing two main functional domains: a methyl-CpG binding domain (MBD) mediating the preferential binding of MeCP2 to methylated CpGs and a transcription repression domain (TRD) capable of repressing transcription through different molecular mechanisms, including the recruitment of histone deacetylase activities to methylated DNA^2^. As of today, MeCP2 is subdivided into five main structural domains corresponding to an N-terminal domain (residues 1–78), the MBD (residues 79–162), the intervening domain (ID; residues 163–206), the TRD (residues 207–310) and the C-terminal domain (residues 311–486) [amino acid numbers refer to the human E2 isoform]. The functional relevance of all these domains, with the exception of the N-terminal domain, can be inferred by their frequent association with pathological missense mutations^3^. Furthermore, the ID and the TRD appear as the domains of MeCP2 that are most commonly involved in several and often labile protein/protein interactions^3^,^4^.

The structural complexity of MeCP2 fits well with its versatile functionality. In mature neurons, where MeCP2 abundance corresponds roughly to one molecule every two nucleosomes, the protein can serve as an alternative linker histone, organizing a specialized chromatin structure that dampens transcriptional noise^5^. MeCP2 can also activate gene transcription possibly through its interaction with CREB1^6^. Additionally, MeCP2 has been proposed to directly affect splicing^7^,^8^, miRNA biogenesis^9^ and centrosomal functions^10^. Post-translational modifications (PTMs) of MeCP2 are likely to generate and regulate this functional versatility. Indeed, mass spectrometry analyses have identified several phosphorylation sites. In neurons, phosphorylation events occur under basal conditions and/or in response to neuronal activity^11^,^12^. The addition of a negatively charged phosphate group can dramatically impact protein functions. Consequently, MeCP2 phosphorylation might affect its sub-nuclear localization^13^. However, immunofluorescence studies performed with phospho-specific antibodies and/or generating specific phospho-defective mutants of MeCP2 failed in identifying an altered intracellular distribution of MeCP2. Further, we still lack insights into the molecular consequences of S421 and S80 phosphorylation of MeCP2, which represent the better-studied MeCP2 post-translational modifications. Of relevance, data obtained from primary neurons led to propose that the neuronal activity dependent phosphorylation of S421 induces the detachment of MeCP2 from specific promoters^14^. However, these results were challenged by a more recent *in vivo* study demonstrating that neuronal depolarization does not alter MeCP2 binding to several promoters. Moreover, the S421A phospho-defective derivative of MeCP2 shows a chromatin distribution that overlaps with that of the wild-type (WT) protein^15^. To conclude, no data have so far been able to demonstrate an effect of MeCP2 phosphorylation on its binding to target sequences or chromatin. In contrast, it has been demonstrated that Threonine 308 (T308) phosphorylation interferes with the interaction of MeCP2 with the corepressor complex NCoR, therefore reducing its repressive activity^16^. Importantly, a phospho-defective *Mecp2*^T308A/y^ knock in mouse line exhibits a large spectrum of neurological symptoms highly phenocopying several mouse models of *Mecp2* disorders^16^. Additionally, it has been proved that MeCP2 phosphorylation impacts dendritic arborization, spine maturation and generally the development and function of the nervous system^11^,^12^. All these observations highlight the relevance of studying the function and regulation of MeCP2 phosphorylation for advancing our comprehension of RTT and *MECP2* related disorders; in particular, we have proposed to start focusing on sites that are evolutionally conserved and/or have been found mutated in patients^12^.

Most studies have so far focused on residues located within the MBD or the TRD. However, several were the rationales for studying phosphorylation of the ID. We have already proposed this protein region to be a frequent target of PTMs^12^. Indeed, out of 44 residues, 7 have been predicted to be subject of phosphorylation in human and experimental evidence is present for 3 (S164, S166 and S178) in mouse (Fig. 1a). K171 has been recently proved to be acetylated^17^, whereas the ID has been demonstrated as a major site of poly(ADP-rybosyl)ation^18^. Both these PTMs highly impact on MeCP2 functions: K171 acetylation modulates the interaction of MeCP2 with ATRX and HDAC1, while (ADP-rybosyl)ation reduces the capability of MeCP2 to cluster heterochromatin^17^,^18^. By analyzing evolutional conservation, we noticed that S164 and S166 have been maintained from *X. laevis* to humans (Fig. 1a). Although none of these sites has been found mutated in *MECP2*-related disorders, S164 and S166 are proximal to a region of the methyl binding domain that is often mutated in patients; furthermore, a close by pathological mutation, R167W, has recently been linked to intellectual disability and autism in males (see Fig. 1a^19^). S164 is particularly interesting since it has been found modified in neuronal tissues; in fact, its phosphorylation was identified by mass spectrometry analysis of immuno precipitated MeCP2 from normal and epileptic rodent brains^20^. Considering all above, we decided to characterize this PTM of MeCP2.

Our studies demonstrate that S164 phosphorylation is the first post-translational modification of MeCP2 that globally affects its sub-nuclear localization and chromatin affinity. In neurons S164 phosphorylation is high in abundance and developmentally regulated; importantly, its proper regulation appears to be necessary for neuronal maturation. The study of this phosphorylation event allowed revealing that MeCP2 dramatically changes its affinity for chromatin along brain maturation.

## Results

### In brain phosphorylation of Mecp2 at S164 is developmentally modulated

**Figure 1:**
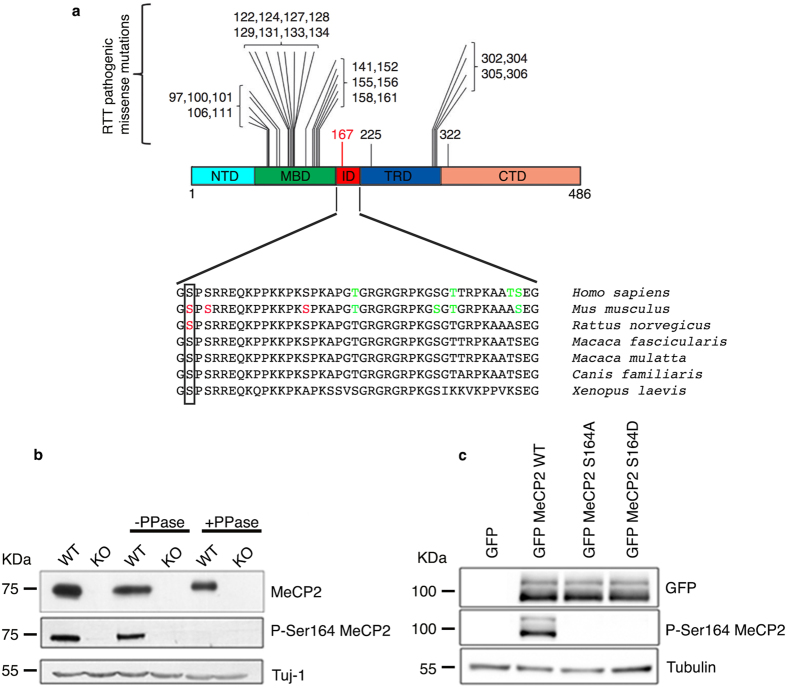
Development of an MeCP2 S164 phospho-specific antibody. **(a)** (Adapted from^16^) Schematic illustration showing the localization of frequent pathogenic missense mutations within MeCP2 domains. The recently identified R167 W pathogenic mutation is indicated in red. Lower part shows alignment of the ID (aa 163–206) from *H. sapiens* to *X. laevis*. Box shows the conserved S164. Red residues represent experimentally determined phosphorylated sites; in green are indicated phosphorylated amino acids predicted by GPs 2.0 and NetPhos 2.0^12^. (**b)** Total brain lysates were prepared from adult WT and KO mice, treated or not with λ phosphatase and analyzed by WB using antibodies against MeCP2 and P-S164 MeCP2. 30 μg of extract were loaded in each lane. Neuronal specific β III tubulin (Tuj1) was used as loading control. **(c)** Extracts from HEK293T cells expressing GFP-MeCP2 or its S164A and S164D derivatives were analyzed by WB with antibodies against P-S164 MeCP2 or total MeCP2. 20 μg of total lysates were loaded in each lane. Tubulin was used as loading control.

To study S164 phosphorylation we raised a polyclonal phospho-site-specific antibody (anti P-S164). Western blotting of WT and *Mecp2-*null brain lysates clearly indicates that the anti P-S164 specifically recognizes Mecp2 in a phosphorylation dependent manner (Fig. 1b). We also tested HEK293T cell lysates expressing exogenous GFP-tagged WT MeCP2 or its phospho-defective (S164A) or phospho-mimetic (S164D) derivatives; although all derivatives of MeCP2 are efficiently expressed, the anti P-S164 antibody fails to recognize both mutant versions of MeCP2 (Fig. 1c). These results demonstrate that the anti P-S164 antibody specifically recognizes the S164 phospho-isoform of MeCP2.

**Figure 2:**
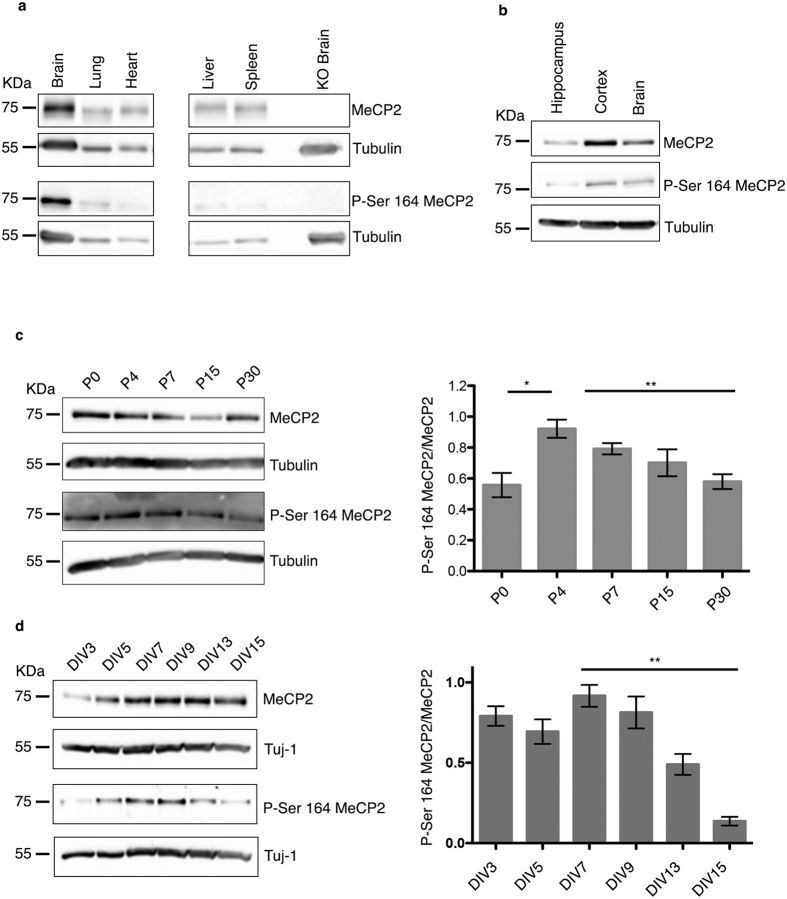
In brain phosphorylation of MeCP2 at S164 is abundant and developmentally regulated. **(a,b)** 60 μg of total brain and tissue lysates (**a**) and 30 μg of total brain or brain area lysates (**b**) were subjected to WB and incubated with antibodies specific to P-S164 MeCP2 or total MeCP2. **(c)** Brains at different ages were lysed and probed with antibodies against MeCP2 and P-S164 MeCP2. Tubulin was used as loading control. The graph represents the ratio between P-S164 MeCP2 and total MeCP2 and was obtained by integrating three independent experiments (mean ± S.E.M *p = < 0.05, **p = < 0.01, ***p = < 0.001, one way ANOVA). **(d)** Lysates of primary hippocampal neurons at the indicated days *in vitro* (DIV) were probed with antibodies against MeCP2 or P-S164 MeCP2. Tuj1 was used as loading control. The graph represents the ratio between P-S164 MeCP2 and total MeCP2 and was obtained by integrating three independent experiments (mean ± S.E.M *p = < 0.05, **p = < 0.01, ***p = < 0.001, one way ANOVA).

By investigating the distribution of P-S164 Mecp2 in adult mouse tissues, we found that, despite some expression in non neuronal tissues, P-S164 Mecp2 is abundant in brain (Fig. 2a).

Although several studies, including ours presented in Fig. 2b, indicate that MeCP2 phosphorylation is often regulated in different brain regions, almost no information is so far available regarding the regulation of these post-translational modifications during brain development. To understand whether S164 phosphorylation is constitutively or dynamically expressed, we compared its levels in the maturing mouse brain with those of total Mecp2. Remarkably, P-S164 Mecp2 is low in abundance at P0, peaks around P4, and gradually decreases at later stages of development (Fig. 2c). Similarly to the maturing brain, the expression of P-S164 Mecp2 is regulated also during neuronal differentiation *in vitro*, reaching a peak after 7–9 days of culture and dramatically diminishing with maturation (Fig. 2d). The peculiar expression profile of P-S164 Mecp2 expression evokes its possible involvement in post-natal neuronal maturation.

Thus, to the best of our knowledge, this is the first report of a phospho-isoform of Mecp2 that is developmentally regulated in brain.

### S164 phosphorylation impairs the affinity of MeCP2 for DNA independently on the methylation status

Two complementary approaches were used to reveal whether S164 phosphorylation generally affects Mecp2 binding to chromatin/heterochromatin and, consequently, its sub-nuclear distribution.

**Figure 3:**
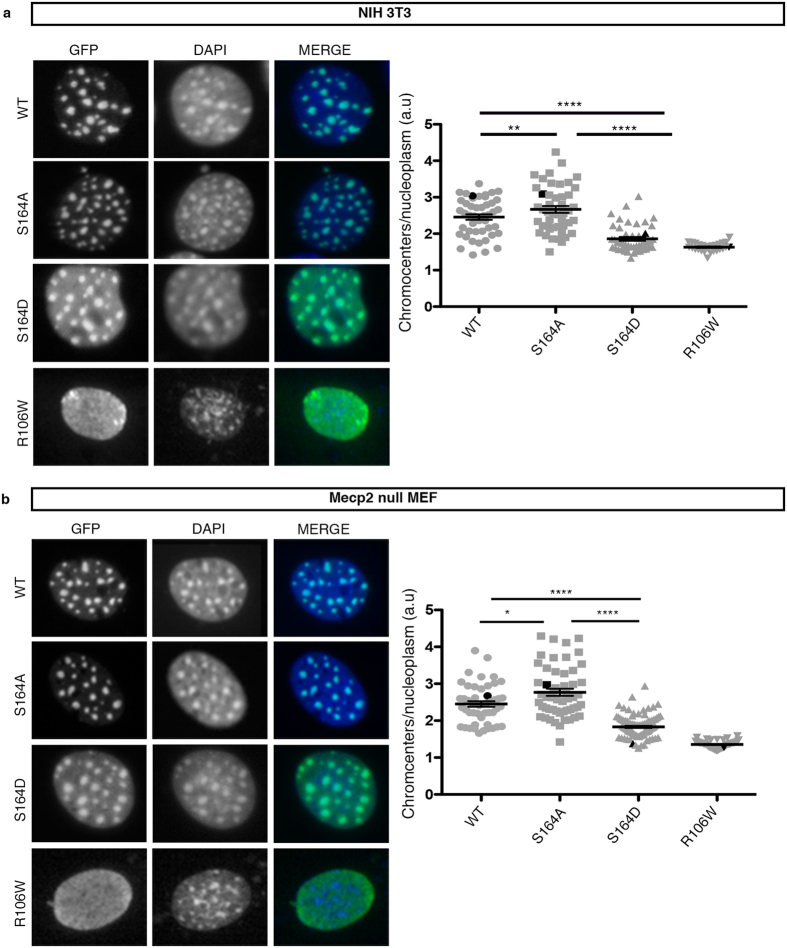
MeCP2 phosphorylation at S164 affects the accumulation at heterochromatic foci. **(a,b)** Representative images from NIH3T3 (**a**) and *Mecp2-*null mouse embryonic fibroblasts (MEF) (**b**) transfected with GFP-MeCP2 WT or its S164A and S164D derivatives. Immunofluorescence was performed using antibodies against GFP and DAPI. The DNA binding-defective R106W mutant was used as control. Graphs show the ratio between fluorescence at chromocenters and at nucleoplasm (mean ± S.E.M). Each dot represents a single cell. Black dots highlight the representative cells shown in each panel. (NIH3T3 WT *n* = 41, S164A *n* = 53, S164D *n* = 101) (MEF WT *n* = 46, S164A *n* = 49, S164D *n* = 50). (*p = < 0.05, **p = < 0.01, ***p = < 0.001, ****p = < 0.0001, one way ANOVA).

Based on the accumulation of Mecp2 at the highly methylated mouse pericentromeric heterochromatin, we first used epifluorescence microscopy to compare the sub-nuclear localization of the exogenously expressed GFP-MeCP2 WT, S164A and S164D derivatives (Fig. 3). The MeCP2 R106W RTT mutant was included in this assay because of its inability to bind methylated DNA and to accumulate on heterochromatic foci^21^,^22^. NIH3T3 mouse fibroblasts (Fig. 3a) and *Mecp2-*null mouse embryonic fibroblasts (MEFs; Fig. 3b), which offer the obvious advantage of not expressing any competing endogenous Mecp2, were used as cell models. The heterochromatic accumulation of the different GFP-fusion proteins was measured as the ratio of the mean intensity of chromocenters versus nucleoplasm (Fig. 3; right panels). Perfectly matching results were obtained with both cell lines. Indeed, WT GFP-MeCP2 colocalizes with the DAPI-positive heterochromatic foci, whereas the R106W mutant shows a largely diffuse localization. Remarkably, the distribution of the phospho-mimetic S164D derivative resembles that of the R106 W mutant by having an increased intensity outside the chromocenters. Accordingly, a statistically significant reduction in the chromocenter accumulation was measured. As further indication that S164 phosphorylation reduces the affinity of Mecp2 for methylated heterochromatin we observed that the S164A phospho-defective derivative, even if resembling WT MeCP2, shows a significantly increased accumulation at heterochromatic foci in both cell types.

**Figure 4:**
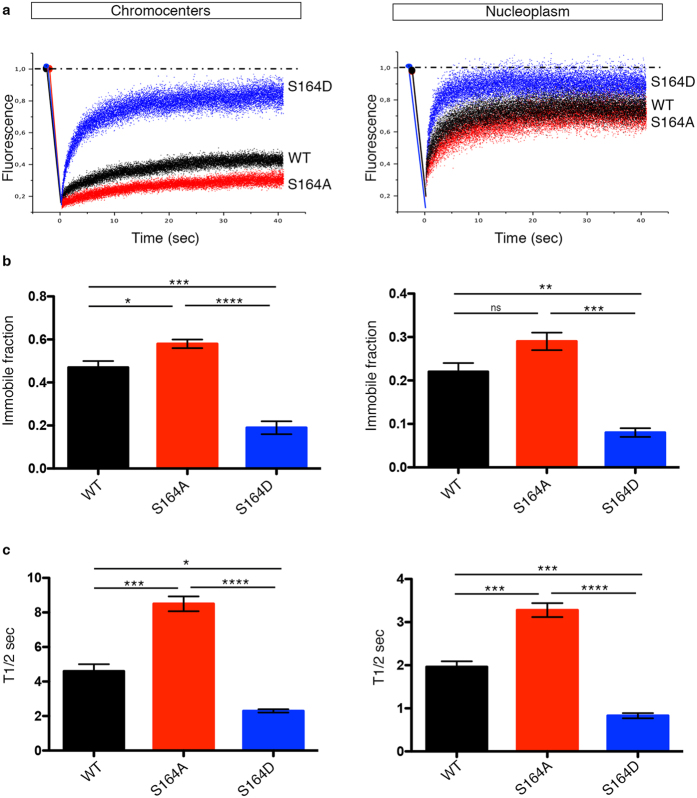
P-S164 MeCP2 shows a minor binding to chromatin. **(a)** Relative mobility of MeCP2 WT, S164A, and S164D in chromocenters (left panel) and nucleoplasm (right panel) of NIH3T3 cells. Representative quantification of the signal intensity: blue (S164D), red (S164A), black (WT). **(b,c)** Summary of average values for immobile fraction (**b**) and T1/2 (**c**) (mean ± S.E.M) measured at chromocenters (left panel) and nucleoplasm (right panel) in cells transfected with GFP-MeCP2 WT (black), S164A (red) and S164D (blue) derivatives (WT *n* = 7, S164A *n* = 9, S164D *n* = 8). (*p = < 0.05, **p = < 0.01, ***p = < 0.001, ****p = < 0.0001, one way ANOVA).

Few groups, including ours, have used fluorescence recovery after photobleaching (FRAP) to study chromatin-binding kinetics of MeCP2 *in vivo*^21^,^23^,^24^. All reports demonstrated that MeCP2 is a mobile protein that, although tightly bound to chromatin, continuously exchanges with the nucleoplasm. A fraction of the protein, however, is irreversibly bound to chromatin. Furthermore, by comparing the recovery of fluorescence at chromocenters with respect to the outside of the heterochromatic foci (nucleoplasm), it has been established that MeCP2 binding is stronger and less reversible in the areas of higher methylation^21^. Similarly, we tested the mobility and chromatin binding of WT GFP-MeCP2 or its phospho-defective and –mimetic derivatives in NIH3T3 cells. Bleaching was performed for 300 milliseconds along a strip (strip-FRAP) covering simultaneously both chromocenter and nucleoplasm regions. A representative recovery of fluorescence after photobleaching for both chromatin fractions of GFP-MeCP2 WT, phosho-mimetic S164D and phospho-defective S164A is indicated in Fig. 4a. We consistently found that WT GFP-MeCP2 shows a stronger binding to heterochromatin (left panels) with a concomitantly ampler immobile fraction than to euchromatin (the nucleoplasmic fraction; right panels). In accordance with Fig. 3, our live-cell kinetic data indicate that the phospho-mimetic S164D derivative interacts only very transiently and with low affinity with both chromatin fractions, whereas the phospho-defective S164A derivative shows the opposite trend and has an overall stronger affinity for chromatin. In fact, the phospho-defective MeCP2 isoform exhibits a statistically significant increase in both the recovery time and amplitude of the immobile fraction.

**Figure 5:**
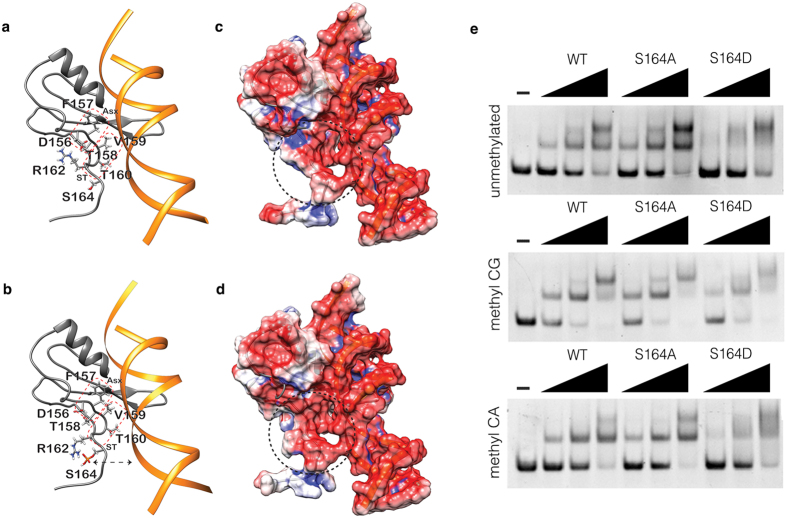
P-S164 MeCP2 shows a more dynamic binding to DNA. **(a)** Ribbon representation of the human MBD model in complex with a short DNA fragment. The residues of the Asx and ST structural motifs are reported in stick together with R162 and S164 residues that are not directly involved in the formation of the two motifs. MBD and DNA structures are colored in grey and orange, respectively. Asx and ST motifs are highlighted with a red dotted square. **(b)** As in panel a, with the addition of S164 phosphorylation. The black dashed arrow represents the electrostatic repulsion effect between the phosphate group of S164 and the DNA backbone. **(c)** Representation of the electrostatic potential mapped on the molecular surface of the MDB-DNA complex in the absence of phosphorylation. The red and blue colours represent the negatively and positively charged regions respectively. The black dashed circle underlines the C-terminal region that differs in the presence or absence of S164 phosphorylation. **(d)** As in c but in the presence of S164 phosphorylation. **(e)** Representative EMSAs showing binding of MeCP2 WT, S164A and S164D peptides to an unmethylated (upper panel), CpG methylated (middle panel) and CpA methylated (lower panel) probe.

To obtain molecular insights regarding the above results, we have modeled the structure of the last portion of the crystal structure of the human MBD-ID (aa 163–170) complexed with a methylated *BDNF* sequence^25^ (Fig. 5a); S164 phosphorylation has also been modeled (Fig. 5b). The C-terminal region of the MBD is characterized by the presence of two structural motifs, Asx and ST^25^. The Asx motif (^156^DFT^158^) forms a turn stabilized by a hydrogen bond between the main chain of T158 and the side chain of D156 (Fig. 5a). The ST motif (^158^TVTG^161^) stabilizes the hydrogen bonds established by the hydroxyl group of T158 with the surrounding residues; this region directly contacts the DNA through V159 (Fig. 5a). Of note, R106 is a key residue for the interaction with DNA^25^ and the orientation of its side chain is stabilized by the peculiar structural arrangement of the Asx and ST motifs^25^.

The three-dimensional models reported in Fig. 5a,b. in the absence and presence of S164 phosphorylation respectively, have been subjected to structure regularization through an energy minimization process. Importantly, S164 phosphorylation leads to a rearrangement of the side chains of the residues belonging to the Asx and ST motifs (Fig. 5b). Upon phosphorylation, the side chain of R162, which is important for the stabilization of the interaction between D156 and T158 (Fig. 5a), changes its orientation, and establishes an electrostatic interaction with the phosphate group bound to S164 (Fig. 5b), therefore modifying the structure of the Asx and ST motifs. Figure 5c,d report the electrostatic potential mapped on the surface of the models after energy minimization. The electrostatic potential of the region surrounding the phosphorylation is more negative with respect to the same region without the modification, thus altering the distribution of the charges in the C-terminal portion of the MBD. This increased negative charge on the protein surface in contact with DNA could cause a repulsive effect with the negatively charged DNA backbone, therefore decreasing the interaction strength between the two macromolecules.

The validity of these structural predictions has been tested analyzing by electrophoretic mobility assays (EMSA) the capacity of MeCP2 fragments (aa 74–172) to bind methylated (mCG; mCA) and unmethylated DNA probes that have already been used^26^. Of note, these polypeptides lack one of the two conserved high-mobility group like AT-hook motifs of MeCP2 (aa 185–197) that, although still devoid of experimental significance, is considered a protein domain binding the minor groove of AT-rich DNA^27^. As shown in Fig. 5e, both the WT and S164A polypeptides show largely overlapping DNA binding properties, characterized by a subtle but consistently higher affinity for DNA methylated on CG. On the contrary, the S164D polypeptide produces a smeared signal, although showing a withdrawal of the free probe comparable to that of the WT peptide. This suggests that the phospho-protein, in accordance with the *in silico* reconstruction, dynamically binds and detaches from the probe; the absence of stable DNA/protein complexes limits the formation of discrete shifted bands.

### P-S164 MeCP2 shows altered nucleosome binding

**Figure 6:**
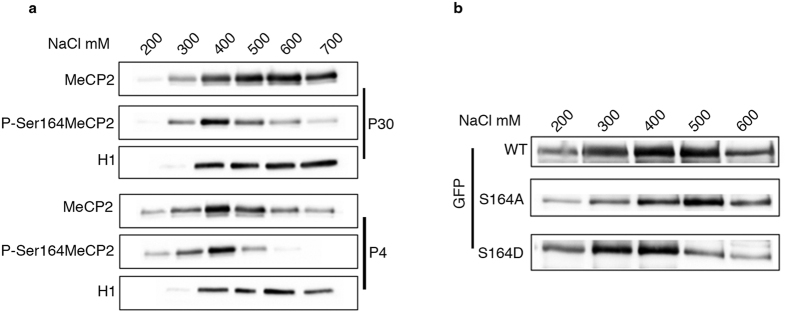
P-S164 MeCP2 represents a more soluble fraction of MeCP2 in brain. **(a)** Western blot showing salt extraction of MeCP2 and P-S164 MeCP2 with increasing concentrations of NaCl from P30 and P4 mouse brains (P30 *n* = 4, P4 *n* = 3). H1 was included as a control of the salt extraction. **(b)** Salt extraction of GFP-MeCP2 and its S164A and S164D derivatives with increasing concentrations of NaCl from transfected NIH3T3 cells (*n* = 3).

Our data suggest that by regulating the phosphorylation state of S164, cells modulate the global affinity of MeCP2 to chromatin and its distribution within the nucleus. We proceeded analyzing whether S164 phosphorylation might similarly affect Mecp2 binding to chromatin *in vivo*. Through salt extraction experiments, we compared P-S164 Mecp2 solubility with respect to that of total Mecp2. Histone H1 withdrawal was used as reference (Fig. 6a). We tested P4 and P30 mouse brains respectively representing high and low levels of P-S164 Mecp2 (Fig. 2c). As reported^28^,^29^, in mature brain (P30) Mecp2 shows a high affinity for chromatin, being mainly extracted between 500 and 700 mM NaCl. In accordance with previous results, P-S164 Mecp2 is more soluble; indeed, it is mainly extracted at 400 mM NaCl (Fig. 6a, upper panel). At P4, the increased solubility of the MeCP2 phospho-isoform is confirmed; strikingly, the solubility of total Mecp2 is dramatically increased and the majority of the protein is extracted at 400 mM NaCl as P-S164 Mecp2 (Fig. 6a, lower panels). We confirmed these data performing salt extraction experiments with NIH3T3 cells expressing GFP-MeCP2 or its phospho-mimetic/defective derivatives (Fig. 6b). Our results demonstrate that WT MeCP2 and its S164A derivative are less soluble than the S164D protein, confirming that the presence of a negative charge on S164 reduces MeCP2 affinity for chromatin.

**Figure 7:**
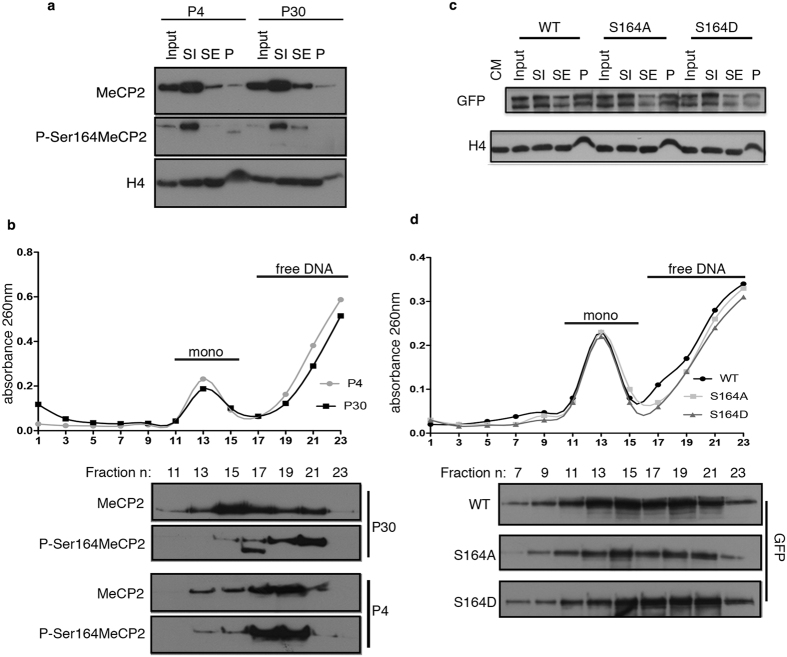
P-S164 MeCP2 is not associated with mononucleosomes in brain. Brain and cell chromatin was subjected to MNase digestion to obtain SI, SE and P chromatin fractions. SI contains histone H1-depleted mononucleosomes; the SE includes H1-containing mononucleosomes and poly-nucleosomes whereas the P fraction represents chromatin resistant to MNase digestion. **(a,b)** Western blots of nuclear MeCP2, P-S164 MeCP2 and H4 partitioning in the SI, SE and P fractions obtained from digested P4 and P30 brain chromatin (**a**). Sucrose gradient fractionation of the SI chromatin fractions (**b**). The graph depicts the DNA absorbance (260 nm) profile of the numbered fractions collected along the gradient. The lower panel shows the western blot analysis using MeCP2 and P-S164 MeCP2 antibodies. **(c,d)** Western blots of nuclear WT GFP-MeCP2, S164A and S164D partitioning in the SI, SE and P chromatin fractions of transfected HEK293T cells (**c**). Sucrose gradient fractionation of the SI chromatin fractions (**d**). The lower panel shows the western blot analysis using GFP antibody.

The chromatin binding properties of Mecp2 probably depend on its affinity for nucleosomal and naked DNA. We used micrococcal nuclease (MNase) digestion to fractionate P4 and P30 brain chromatin (see Materials and Methods). It has been described that SI corresponds to a histone H1-depleted soluble fraction; the SE includes H1-containing mononucleosomes and nucleosome oligomers whereas the P fraction represents highly compacted chromatin resistant to MNase digestion^29^,^30^. As previously reported, we found that in adult brain most Mecp2 co-fractionates with SI. The same distribution is observed in P4 brains; P-S164 Mecp2 follows the overall protein distribution (Fig. 7a).

SI samples were fractionated on sucrose gradients to describe the distribution of P-S164 Mecp2 amid chromatin. Figure 7b shows that mononucleosomes peak between fractions 11–15 (upper panel). The western blot demonstrates that in adult brain the majority of total Mecp2 co-fractionates with mononucleosomes, although a fraction of the methyl-binding protein sediments with nucleosome-free DNA (fractions 17–23). P-S164 Mecp2 shows a different profile with most of it co-sedimenting with free DNA. Importantly, in immature brain (P4), both total Mecp2 and its phospho-isoform show identical sedimentation profiles.

These data reinforce our results showing that the chromatin-binding properties of Mecp2 are extensively modified along brain maturation and that these changes are at least partially mediated by its differential phosphorylation. To confirm that phosphorylation of S164 significantly affects Mecp2 distribution among nucleosomes, the same assay was performed on 293T cells transfected with WT GFP-MeCP2 and its S164-mutated derivatives. The accumulation of all three exogenously expressed proteins in the SI fraction was confirmed (Fig. 7c). However, it is noticeable that the phospho-mimetic MeCP2 derivative is less represented in the insoluble P fraction, thus confirming its decreased affinity for chromatin. Sucrose gradient sedimentation of the SI fractions showed that a large fraction of the S164D derivative associates with nucleosome-free DNA, whereas the unphosphorylatable mutant mainly co-sediments with nucleosomes (Fig. 7d).

### S164 phosphorylation controls nuclei dimension and dendritic patterning *in vitro*

**Figure 8:**
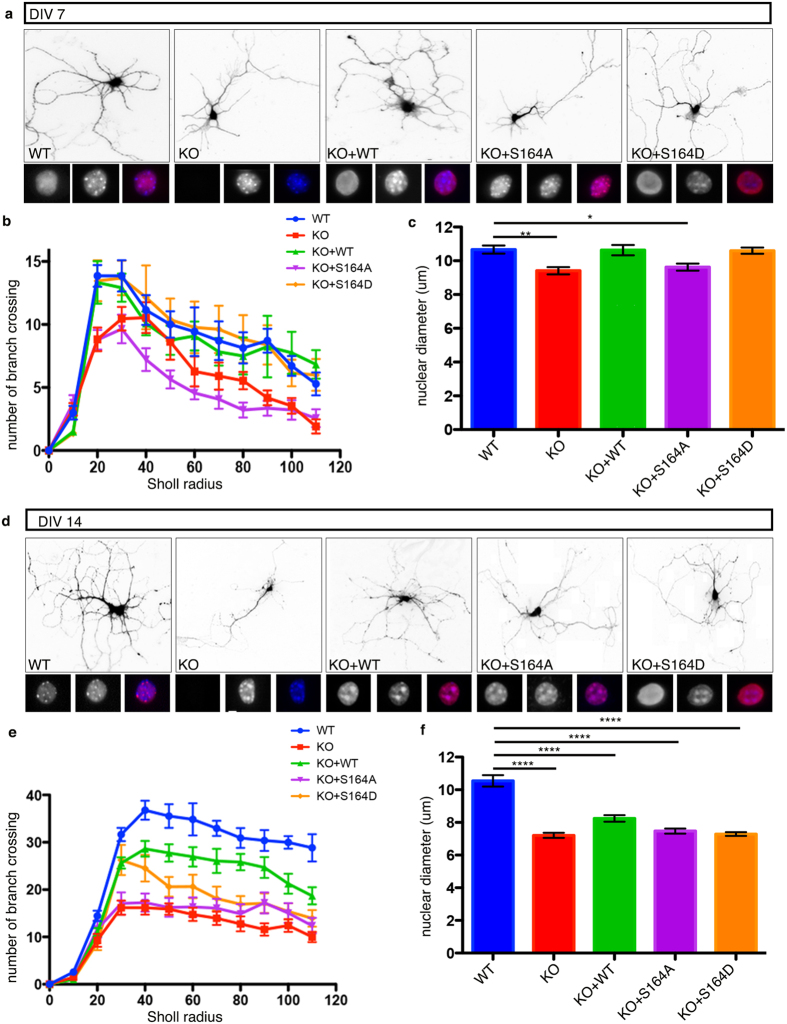
A dynamic phosphorylation of MeCP2 at S 164 is necessary to ensure proper neuronal dendritic branching and nuclear size. **(a,d)** Representative images from DIV7 (**a**) and DIV 14 (**d**) neurons prepared from WT or *Mecp2* KO E15 cortices. For each condition, under the GFP panel, the three squares show magnification of nuclei stained respectively with: MeCP2 (left), DAPI (centre); merged image is on the right. KO neurons were infected at 0 DIV with MeCP2 WT (KO+WT) or the S164A (KO+S164A) and S164D (KO+S164D) derivatives. **(b,e)** Quantification of dendritic branch complexity by Sholl analysis at DIV7 (**b**) and 14 (**e**) (data are presented as mean ± SEM) (See Supplementary information Fig. S2 for statistical analyses). **(c,f)** Quantification of nuclear diameter at DIV7 (**c**) and 14 (**f**) (data are represented as mean ± SEM, *p = < 0.05, **p = < 0.01, ***p = < 0.001, ****p = < 0.0001, one way ANOVA) (three animals per genotype at least 20 neurons per condition).

We have previously demonstrated that Mecp2 affects nuclear size and neurite length in cultured neurons^31^. A role for S421 Mecp2 phosphorylation in dendritic growth of primary neurons has also been established^32^. We exploited these Mecp2-related phenotypes to understand whether S164 phosphorylation is involved in neuronal maturation. Cultures were assayed at DIV7, when neuronal maturation is progressing and at DIV14, when functional synapses are present^33^. *Mecp2-*null neurons were infected with lentiviruses expressing the WT or the mutated derivatives of S164; the concomitant expression of GFP from the bicistronic cassette permitted to visualize neuronal morphology (Fig. 8). WT neurons infected with GFP expressing viruses were used as control. The expression of all derivatives was verified (Fig. S1), demonstrating that in mature neurons exogenously expressed MeCP2 is roughly 2.5 times more abundant than the endogenous protein.

By immunofluorescence we analyzed the subnuclear distribution of the exogenously expressed proteins. In line with mouse cerebral cortex^34^, although well-defined heterochromatic foci are present in DIV7 neurons, neither endogenous nor exogenous WT MeCP2 accumulates on them (Fig. 8a); the accumulation on pericentromeric heterochromatic is instead well recognizable in more mature neurons (Fig. 8d). In accordance with previous publications^24^, at DIV14, MeCP2 overexpression causes heterochromatin clustering leading to larger and less numerous DAPI positive foci (Fig. 8d; KO+WT). Importantly, the S164A derivative accumulates on heterochromatic foci already at DIV7, whereas the S164D derivative is diffused at both stages of neuronal maturation. We confirmed that at DIV7 *Mecp2-*null neurons display smaller nuclei (Fig. 8c) and an evident reduction in the number of neurites longer than 80–100 μm (Fig. 8b; for detailed measurements and statistical analyses see Fig. S2). WT and S164D derivatives normalized both the impaired dendritic branching and nuclear size. Conversely, neurons expressing the S164A mutant resembled the KO cells (8b,c). By repeating the analyses at DIV14, we found that *Mecp2-*null neurons have an exacerbated defect both in nuclear dimension (Fig. 8f) and dendritic branching (Fig. 8e). Neurons overexpressing WT MeCP2 show a dendritic phenotype that, although not reaching statistical significance, is normalized towards that of WT neurons. This result is in line with previous data suggesting that increased MeCP2 levels become more detrimental as maturation proceeds^35^. Overexpression of the S164D or S164A mutants did not show amelioration of the neuronal phenotypes. The capacity of the phospho-mimetic mutant to normalize the morphological defects of immature but not mature *Mecp2-*null neurons suggest that dephosphorylation of S164 is necessary to accomplish proper neuronal maturation. These experiments suggest the importance of the dynamic phosphorylation of S164, an event that is needed during early neuronal maturation but that must be dynamically regulated for terminal differentiation.

## Discussion

Several biological processes have been linked to MeCP2 including activation and repression of transcription, regulation of global chromatin structure, control of alternative splicing and the biogenesis of non-coding RNAs^2^,^3^. Further, we demonstrated that MeCP2 affects protein synthesis^36^ and centrosomal functions^10^. These findings imply that MeCP2 is a multifunctional protein, whose numerous activities still need to be unraveled. The intrinsically disordered structure of MeCP2 and its several PTMs have been suggested to generate and regulate this functional versatility^31^,^37^. Several reports have proposed that differential phosphorylation of MeCP2 is a key mechanism by which the protein modulates its affinity for partners and/or DNA^11^,^12^. However, most of the MeCP2 PTMs studied so far involve almost exclusively the MBD/TRD regions of the protein and phosphorylation sites within the ID have never been characterized.

The ID consists of a short basic region with an amino acid composition similar to HMGA1^38^. The high density of basic residues in the ID appears to positively influence the association of MeCP2 with the negatively charged DNA backbone. Indeed, FRAP analyses proved that the MBD is necessary and sufficient for targeting MeCP2 to heterochromatic foci, whereas the ID is crucial for its proper chromatin association. Deletion of the ID accelerates the binding kinetics of MeCP2 without influencing its subnuclear localization^23^. *In vitro,* the ID and TRD contribute to the interaction of MeCP2 with DNA and nucleosomes^39^. It has thus been proposed that MeCP2 organizes chromatin structure through homo- and hetero associations, largely mediated by its ID and TRD^40^. The relevance of the ID is further highlighted by the recent identification of two missense mutations (p.Gly185Val and p.Arg167Trp) in families affected by intellectual disability^19^. Missense mutations in the ID are inherited from asymptomatic mothers with skewed X-chromosome inactivation; in males they cause intellectual disability and not encephalopathy or RTT^19^,^41^. We emphasize that these *MECP2* alterations either do not appear in the RettBase syndrome database (http://mecp2.chw.edu.au; p.Gly185Val) or are still reported as of uncertain significance. Interestingly, the database reports five additional pathogenic mutations (P172; P173; P179; A181 and P199) affecting the ID region.

Considering all above and the fact that S164 was the only ID residue found phosphorylated in rodent brain^20^, we used *in silico* modeling to add structural information useful to understand the impact of S164 phosphorylation on MeCP2 binding to DNA. As reported by the X-ray structure, the C-terminal region of the MBD has a crucial role in the interaction with DNA^25^. S164 phosphorylation is expected to have two main effects on the adjacent MBD: a destabilization of the structure of the Asx and ST motifs (mediated by an electrostatic interaction with R162) and a lower affinity of the MBD for DNA, independently on its epigenetic modifications, caused by the addition of a negative charge at the interaction surface.

The *in silico* predictions have been confirmed by our EMSAs. Indeed, the polypeptide carrying the S164D mutation shifts the DNA probes but forms diffused bands, indicating that the S164D-polypeptide/DNA complex is unstable under these electrophoretic conditions. Expression of the full-length MeCP2 derivatives in cells confirms that the phospho-mimetic mutant is characterized by a more dynamic binding both to euchromatin and heterochromatin, whereas the phospho-defective derivative has a slightly but significantly increased affinity for chromatin. Accordingly, in adult brain, P-S164 Mecp2 dissociates from chromatin at lower ionic strength than global Mecp2, highlighting the impact of this PTM on the affinity of MeCP2 for DNA.

Skene *et al.* demonstrated that Mecp2 is globally bound to the genome in mature neurons, tracking the presence of methylated nucleotides^5^. The authors argued that, in neurons, MeCP2 functions as a chromatin architectural factor dampening transcriptional noise genome-wide, thus rendering the identification of MeCP2 direct targets less relevant. It was suggested that MeCP2 phosphorylation might transiently relieve this global repression and facilitate appropriate gene expression. However, ChIP-seq approaches and/or immunofluorescence experiments used to describe the genomic distribution and/or intracellular localization of specific phospho-isoforms of MeCP2 (P-S421, P-S424, P-S80, P-T308, P-S229)^12^ failed in revealing a global detachment of P-Mecp2 from chromatin. S164 phosphorylation appears to be the first described event of MeCP2 phosphorylation capable of globally affecting its binding to chromatin. This PTM occurs mainly in maturing brains and neurons rather than in mature cells. Importantly, our salt extraction analyses demonstrate that in perinatal brains, when Mecp2 is highly phosphorylated on S164, the methyl-binding protein is also less tightly bound to chromatin. Given the consistency of these data with the overexpression of MeCP2 S164 derivatives in cells, we suggest that the activity of P-S164 MeCP2 is not influenced by its abundance.

Here we report for the first time a phosphorylation event of MeCP2 depending on neuronal development. Moreover, our results provide the first demonstration that the affinity of MeCP2 binding to nucleosomes is significantly influenced by brain maturation reinforcing the novel notion that MeCP2 functions are developmentally regulated. We speculate that, similarly to histones^42^, such regulation is mainly achieved through differential PTMs that altogether generate an MeCP2 code with its counterpart of writer, reader and eraser multiprotein complexes.

Genome-wide chromatin immunoprecipitations have been used to identify whether MeCP2 phospho-isoforms or-derivatives differ in their genomic distribution. No changes have been reported so far^15^. These results might reflect the technical constraints of this approach. Indeed, since MeCP2 is globally bound to the genome, in these types of analyses it is certainly difficult to achieve the necessary sequencing depth to properly quantify any local change. Further, the time of formaldehyde crosslinking affects the capability to capture transient MeCP2-DNA interactions and the field is still missing a gold standard protocol. Considering all above, and the caveat that S164 phosphorylation alters the dynamics of MeCP2 binding to chromatin, we decided to not carry out these studies at present.

When the expression of WT MeCP2 or its phospho-derivatives in Mecp2-null primary neurons was used to study the relevance of S164 phosphorylation, we found that the WT and S164D derivative, but not the S164A mutant, have the capacity to normalize both the nuclear diameter and dendritic arborization in young neurons. This indicates that the mere ability of MeCP2 to bind DNA is not sufficient to rescue phenotypes caused by the lack of the protein and that proper MeCP2 regulation through PTMs plays a crucial role during neuronal development. In mature neurons, we did not observe any rescue with the phospho-mimetic or -defective derivatives. By analyzing overt morphological phenotypes at two different DIVs, we demonstrated that the detrimental effects of MeCP2 overexpression worsen with neuronal maturation, suggesting the need of testing the functional relevance of MeCP2 mutants in immature primary neurons.

To conclude, our data highlight S164 phosphorylation as a developmentally regulated PTM of MeCP2 with a relevant impact on its DNA/chromatin binding properties. Further, they provide support to the hypothesis that MeCP2 PTMs might integrate different signals, which finally lead to adaptive structural and/or transcriptional outputs.

To date, no pathologic mutation has been reported at this site. As a possible explanation, we can assume that additional PTMs synergistically occur with S164 phosphorylation to develop a full functional outcome. This hypothesis fits well with the demonstration that in mouse brain MeCP2 poly(ADP-ribosyl)ation decreases DNA binding affinity and chromatin clustering^18^. Importantly, the main region affected by this PTM was narrowed down to amino acids 106–203. It is also possible that by extending the cohorts of patients screened in molecular testing to include mild neurological symptoms, pathological mutations at S164 will be identified.

## Materials and Methods

### Plasmids

Amplified hMeCP2E1 was cloned into the BamHI site of pEGFPC1 (Clontech). MeCP2-S164A/D mutants were obtained by site directed mutagenesis using the QuickChange XL Site-Directed Mutagenesis Kit (Stratagene). MeCP2 fragments (aa 74–172) were PCR amplified from the pEGFPC1-MeCP2 derivatives (WT, S164A/D) and cloned into pTYB1 vector (NEB). The pLLiGFP was obtained by cloning the iresGFP cassette from pCAGGSiGFP plasmid into pLL3.7 hMeCP2E1 WT, S164A/D were cloned into the BamHI site of the pLLiGFP vector.

All constructs were verified by sequencing.

### Animals

The *Mecp2*-null mouse strain, originally purchased from Jackson Laboratories (003890 B6.129P2(C)-Mecp2<tm1.1Bird>/J), was maintained on a CD1 background and genotyped as described in^31^. Animals were kept in our on site animal room and routinely checked for general health conditions. All procedures were performed in accordance with the European Community Council Directive 86/609/EEC for care and use of experimental animals; protocols were approved by the Italian Minister for Scientific Research and by the San Raffaele Scientific Institutional Animal Care and Use Committee in accordance with the Italian law.

### Generation of phospho-specific antibodies

The anti-MeCP2 S164 phospho-site specific antibody was generated by Diagenode, Inc. Rabbits were immunized with the peptide [NH_2_]-TGRG-pS-PSRRE-[COOH]. The antibody was purified by Covance, Inc, passing the antiserum on a column containing the non-phosphorylated peptide and applying the flow-through to a second column that was conjugated to the phosphorylated peptide.

### Cell culture and transfection

HEK293T and NIH3T3 cells were maintained in DMEM (Sigma-Aldrich) supplemented with 10% FBS, L-glutamine, 100 units/ml penicillin, and 100 μg/ml streptomycin. MEF cultures from WT and *Mecp2*-null mice were prepared from E14 as described in^10^.

Cells were plated and transfected exploiting Lipofectamine^TM^ 2000 (Life Tech.) or with the calcium phosphate method. Cells were collected or fixed 24 hour post-transfection.

### Immunofluorescence

MEF and NIH3T3 cells were fixed with 4% PFA for 20 min at RT, permeabilized and blocked for 1 h with 5% Horse serum, 0.2% Triton-X-100 in TBS. An anti GFP antibody (Roche 11814460001, 1:1000) was added overnight (4 °C). Slides were incubated with the secondary antibody (Anti mouse Alexa Fluor 488) for 1 h at RT and washed. DNA was counter stained with DAPI (Life Tech.). Slides were mounted with ProLong Gold Antifade Reagent (Life Tech.). Images were acquired with a NikonEclipse Ni upright microscope. All images were acquired with identical settings for exposure time. Analysis of fluorescence at chromocenters and in the nucleoplasm was performed using ImageJ (NIH) threshold tool.

### Primary neuronal cultures

Primary neuronal cultures were prepared from mouse embryo brains of either E15 or E17 as described in^31^. Neurons were cultured in Neurobasal medium (Invitrogen) supplemented with 2% B27 (Invitrogen) and glutamine (Sigma Aldrich). 1 × 10^4^ hippocampal neurons were plated on coverslips in 24-well plates and 1 × 10^5^ cortical neurons in 6-well plates, both coated with poly-L-lysine.

### Lentiviral production

HEK293T cells were cotransfected with the packaging vectors pVSV-G, pMDL, pREV and either pll3.7iGFP or pll3.7 hMeCP2iGFP, pll3.7 hMeCP2S164AiGFP and pll3.7 hMeCP2S164DiGFP using CaCl_2_ transfection protocol. Viral particles were harvested 36 h post-transfection and concentrated 250-fold by ultracentrifugation^43^. Viruses were resuspended in PBS and stored at −80 °C. Viral titers were typically 3–6 × 10^6^ pfu/μl. Neurons were infected with lentiviruses at a final concentration of 2.5 ×1 0^6^ pfu/ml for 6 well plates and 2.5 × 10^5^ pfu/ml for 24 well plates at 0–1 DIV. To enhance GFP expression, neurons used for morphological analyses were coinfected with lentiviruses encoding GFP.

### Morphological analysis

Morphological analyses were performed on cortical neurons, DIV7 and 14. Immunofluorescence was performed as described using antibodies against GFP, MeCP2 (Sigma M9317, 1:200) and DAPI. Sholl analysis was performed using the dedicated plugin of ImageJ. Size of neuronal nuclei was evaluated with ImageJ by measuring the DAPI-stained area of each nucleus.

### Protein extraction and western blotting

Mouse tissues and transfected cells were homogenized in lysis buffer (50 mM Tris HCl pH 7.4, 150 mM NaCl, 1% Triton X-100, 2 mM EDTA, 1 mM DTT) supplemented with PhosSTOP (Roche), cOmplete EDTA-free protease inhibitor cocktail (Roche) with a dounce homogenizer. Homogenates were centrifuged at maximum speed at 4 °C for 15 minutes, and the supernatant collected.

Proteins were separated on either a 8 or 10% SDS-PAGE and transferred to nitrocellulose membrane. Membranes were blocked in TBST (10 mM Tris–HCl, pH 8.0, 150 mM NaCl and 0.05% Tween-20) containing 5% milk and incubated with primary antibodies overnight at 4 °C. The following primary antibodies were used: MeCP2 (Sigma M9317, 1:1000), pS164-MeCP2 (1:1000), Tuj-1 (Sigma T2200, 1:10.000), GFP (Roche 11814460001, 1:1000), H1 (GeneTex GTX87506, 1:1000), and H4 (made in house^29^, 1:10.000). After washing filters were incubated with appropriate secondary antibody (Life Technologies) for 1 hour at RT. Detection was performed by enhanced chemiluminescence (EuroClone). For quantitative measurements, autoradiographs were acquired using a G-box Chemi XX6 (Syngene) or scanned. Image analysis was performed with ImageJ (NIH).

### Chromatin fractionation

Brains and transfected cells were homogenized in buffer A (0.25 M sucrose, 60 mM KCl, 15 mM NaCl, 10 mM MES pH 6.5, 5 mM MgCl_2_, 0.5% Triton and PhosSTOP (Roche), cOmplete EDTA-free protease inhibitor cocktail (Roche) and incubated for 10 minutes on ice. Nuclei were collected by centrifugation at 4500 xg for 10 minutes (4 °C). Isolated nuclei were resuspended in buffer B (50 mM NaCl, 10 mM PIPES pH 6.8, 5 mM MgCl_2_, 1 mM CaCl_2_) to a final concentration of 2 mg/ml at A260. Nuclei were digested using MNase (Worthington) at a concentration of 30 U/mg of DNA at 37 °C for 15 minutes. Reactions were stopped by adding EDTA to a final concentration of 20 mM on ice. Digested nuclei were immediately centrifuged at 9000 xg for 10 min at 4 °C to collect S1 supernatant fraction. The remaining pellet was lysed in 0.25 mM EDTA with stirring for 1 hour (4 °C). After centrifugation at 16.000 xg for 10 minutes at 4 °C, the supernatant SE and an insoluble lysis-resistant pellet (P) were produced.

### Sucrose gradient fractionation

The SI supernatant was fractionated on a 5–20% sucrose gradient in 25  mM NaCl, 10  mM Tris pH 7.5 and 5  mM EDTA buffer at 96.000 xg for 21 hours (4 °C) using a Beckman L8–70 M ultracentrifuge and a SW28 swing rotor with no deceleration. 0.5 ml fractions were collected and subjected to western blotting to determine the presence of phosphorylated and total MeCP2.

### Salt extraction

Brains and transfected cells were lysed in 10 mM Hepes pH 7.5, 1.5 mM MgCl_2_, 10 mM KCl, 0.2% NP40, 0,1 mM EDTA, 10% glycerol, PhosSTOP (Roche), cOmplete EDTA-free protease inhibitor cocktail (Roche), on ice, using a potter homogenizer. Samples were incubated 30 min on ice and centrifuged at 2000 xg for 5 min at 4 °C. Nuclear pellets were resuspended in 10 mM Tris pH 7.4, PhosSTOP, cOmplete EDTA-free protease inhibitor cocktail supplemented with 200 mM NaCl. Samples were vortexed and incubated on ice for 20  min, followed by centrifugation at 2000xg for 5 min. Supernatants were collected. The same procedure was repeated for other 5 times, supplementing each time the sample with the same buffer but containing an increased amount of NaCl corresponding to 300, 400, 500, 600 and 700 mM. For western blotting analysis, equal volume of each fraction was loaded on a 10% acrylamide gel.

### Fluorescence recovery after photobleaching

GFP-MeCP2 WT, GFP-MeCP2 S164A and GFP-MeCP2 S164D plasmids were transiently transfected in NIH3T3 cells with Lipofectamine 2000 (Life Technologies). Twenty hours post-transfection cells were analyzed as described in^21^.

### Recombinant MeCP2 purification

An overnight pre-culture of ER2566 cells transformed with a MeCP2(74–172)TYB1 plasmid was diluted 100 times in LB (100 μg/ml ampicillin) and allowed to grow at 37 °C up to 0.6–0.8 OD at 595 nm. IPTG (1mM) induction was performed at 30 °C for 5 hours. Bacteria were collected and lysed in lysis buffer (750 mM NaCl, 20 mM Tris-HCl pH 8, 1 mM EDTA pH 8, 0.1% Triton X100) followed by sonication (15 min 100% amplitude). Supernatant lysates were collected by centrifugation and passed 5 times through a chitin resin (NEB) column. Proteins were eluted by incubating over night at 4 °C the columns in lysis buffer containing 50 mM DTT, and collecting fractions of 200 μl. Eluted polypeptides were dialyzed against 20 mM Hepes, 100 mM NaCl, 10% Glycerol, 0.250 mM EDTA pH 8 and quantified using BCA assay.

### Electrophoretic mobility shift assay

Oligonucleotides probes either unmethylated or containing one methyl CA or a symmetrically methylated CG were annealed by boiling reverse and forward strands at 95 °C for 5 min and then cooling down for at least 4 hours. Sequences were as follows^26^:

Forward 5′- CTAAAGGAAAAGTGA**C**G**C**ACTGTTTTACACCTTGGTTTT-3′

Reverse 5′- AAAACCAAGGTGTAAAACAGTG**C**GTCACTTTTCCTTTAG-3′

Bold C refers to methylated residues.

Increasing amounts of MeCP2 recombinant protein (0, 125, 250, 500 ng) were incubated with 0.1 mg/ml BSA and 200 ng of annealed probe in EMSA buffer (10 mM Tris HCl, 50 mM KCl, 0.5 mM MgCl_2_, 0.1 mM EDTA, 5% glycerol) at RT for 30 min. Samples were loaded on a 6% acrylamide gel in 1X TAE. Electrophoresis was developed at 100 V for 1 h. Gels were stained with ethidium bromide.

### *In silico* molecular modeling

The crystal structure of the hMeCP2 MBD in complex with a methylated DNA sequence from BDNF (PDB ID 3C2I)^25^ has been used as starting structure to model the C-terminal region of the MBD. The last 8 residues (Gly163 to Gln170) were modeled using the I-Tasser web server^44^ while phosphorylation was added using the Sybyl software (www.tripos.com). The structure of both models was regularized using 10.000 steps of the energy minimization protocol applying the steepest descents algorithm. The energy minimization was performed using the AMBER14 software.

The PBEQ-Solver web server^45^ was used for the calculation of the electrostatic potential. The structures obtained after energy minimization were submitted to the server for calculations.

Figures were obtained using UCSF Chimera v. 1.10.1^46^.

## Additional Information

**How to cite this article**: Stefanelli, G. *et al.* Brain phosphorylation of MeCP2 at serine 164 is developmentally regulated and globally alters its chromatin association. *Sci. Rep.* **6**, 28295; doi: 10.1038/srep28295 (2016).

## Supplementary Material

Supplementary Information
