# Author Correction: Brain phosphorylation of MeCP2 at serine 164 is developmentally regulated and globally alters its chromatin association

**DOI:** 10.1038/s41598-021-90392-3

**Published:** 2021-05-26

**Authors:** Gilda Stefanelli, Anna Gandaglia, Mario Costa, Manjinder S. Cheema, Daniele Di Marino, Isabella Barbiero, Charlotte Kilstrup-Nielsen, Juan Ausió, Nicoletta Landsberger

**Affiliations:** 1 San Raffaele Rett Research Unit, San Raffaele Scientific Institute, Milan, Italy; 2 Institute of Neuroscience, National, Research Council (CNR), Scuola Normale Superiore Pisa, Italy; 3 Department of Biochemistry and Microbiology, University of Victoria, Victoria (BC), Canada; 4 Department of Informatics, Institute of Computational Science, Università della Svizzera Italiana, Lugano, Switzerland; 5 Department of Biotechnology and Life Sciences, University of Insubria, Busto Arsizio (VA), Italy; 6 Department of Medical Biotechnology and Translational Medicine, University of Milan, Milan, Italy

Correction to: *Scientific Reports* 10.1038/srep28295. Published online 21 June 2016

This Article contains an error in Figure 1c where the blots for GFP-MeCP2 and P-S164 MeCP2 were inadvertently truncated. In addition, the label for GFP-MeCP2 is incomplete. The correct Figure 1 appears below as Figure 1.

**Figure 1:**
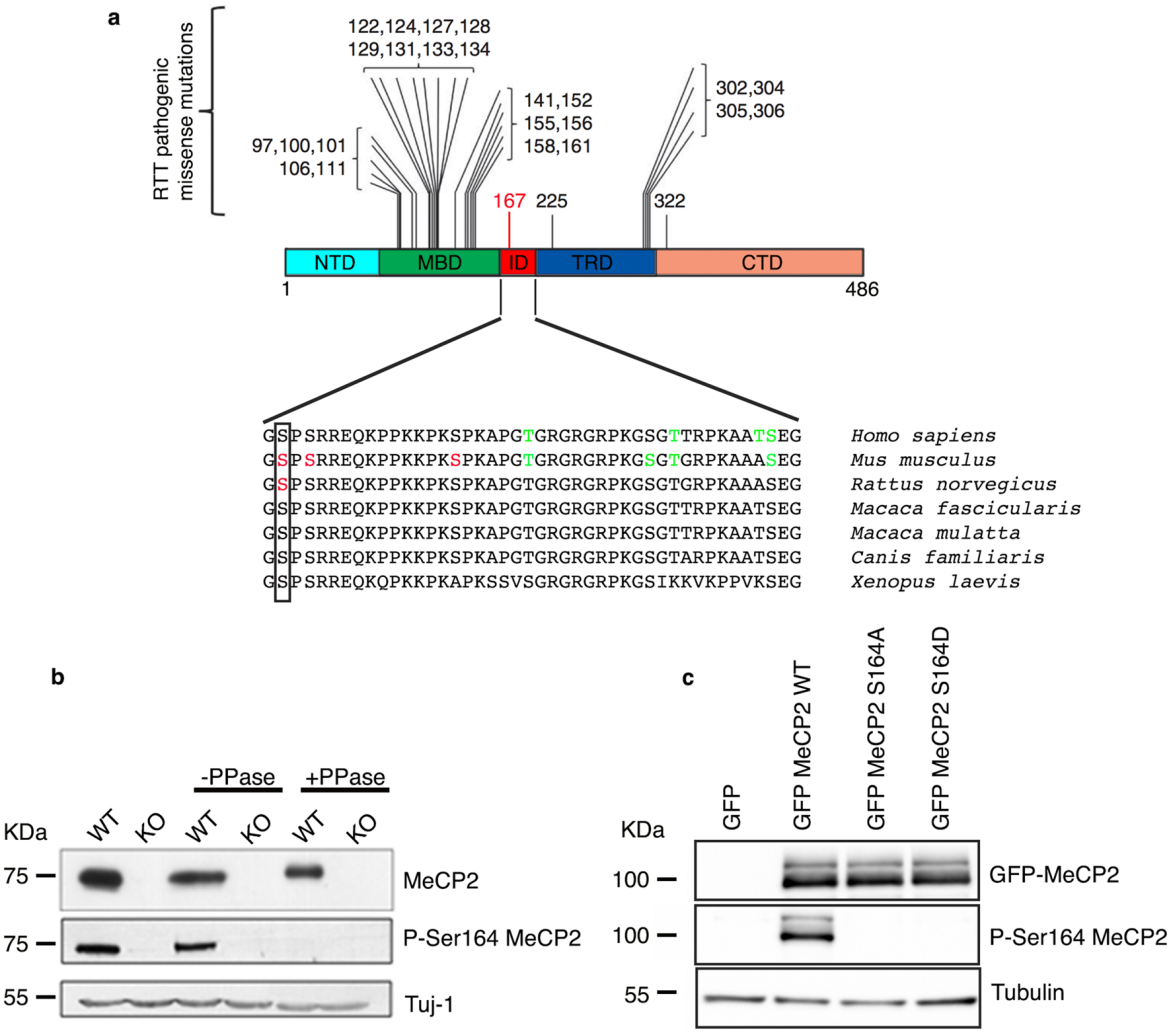
Development of an MeCP2 S164 phospho-specific antibody. (**a**) (Adapted from^16^) Schematic illustration showing the localization of frequent pathogenic missense mutations within MeCP2 domains. The recently identified R167 W pathogenic mutation is indicated in red. Lower part shows alignment of the ID (aa 163–206) from *H. sapiens* to *X. laevis*. Box shows the conserved S164. Red residues represent experimentally determined phosphorylated sites; in green are indicated phosphorylated amino acids predicted by GPs 2.0 and NetPhos 2.0^12^. (**b**) Total brain lysates were prepared from adult WT and KO mice, treated or not with λ phosphatase and analyzed by WB using antibodies against MeCP2 and P-S164 MeCP2. 30 μg of extract were loaded in each lane. Neuronal specific β III tubulin (Tuj1) was used as loading control. (**c**) Extracts from HEK293T cells expressing GFP-MeCP2 or its S164A and S164D derivatives were analyzed by WB with antibodies against P-S164 MeCP2 or total MeCP2. 20 μg of total lysates were loaded in each lane. Tubulin was used as loading control.

Consequently, the accompanying Supplementary Information file is provided below.

## Supplementary Information

Supplementary Information.

