# Supplementary material for: Brain phosphorylation of MeCP2 at serine 164 is developmentally regulated and globally alters its chromatin association
Source: Sci Rep. 2016 Jun 21;6:28295. doi: 10.1038/srep28295 (PMC4915018; doi:10.1038/srep28295)
Supplement: Supplementary Information [file srep28295-s1.pdf]

Supplementary Information for

Brain phosphorylation of MeCP2 at serine 164 is developmentally regulated and globally alters its chromatin association

Gilda Stefanelli[1], Anna Gandaglia[1], Mario Costa[2], Manjinder S. Cheema[3], Daniele Dimarino[4], Isabella Barbiero[5], Charlotte Kilstrup-Nielsen[5], Juan Ausió[3] and Nicoletta Landsberger[*,1,6]

[1]San Raffaele Rett Research Unit, San Raffaele Scientific Institute, Milan, Italy

[2]Institute of Neuroscience, National research council (CNR), Scuola Normale Superiore Pisa, Italy

[3]Department of Biochemistry and Microbiology, University of Victoria, Victoria (BC), Canada

[4]Department of Informatics, Institute of Computational Science, Università della Svizzera Italiana, Lugano, Switzerland

[5]Department of Biotechnology and Life Sciences, University of Insubria, Busto Arsizio (VA), Italy

[6]Department of Medical Biotechnology and Translational Medicine, University of Milan, Milan, Italy

[*]corresponding author

qPCR

Total RNA was harvested using Trizol reagent (Sigma), 1 ml for 200.000 cells following instructions. RNA concentration was quantified with NanoDrop (Thermo Scientific) and its quality verified through gel electrophoresis. 250 ng of RNA were reverse transcribed using the RT^2 First Strand Kit (QIAGEN). qPCRs were performed using SYBR green (Life Tech.) as fluorescent dye.

Used primers were:

hMeCP2 FF 5'-TGGGAAGCTCCTTGTCAAGAT-3'

hMeCP2 Rev 5'-TCGGATAGAAGACTCCTTCACG-3'.

18S FF 5'-GTAACCCGTTGAACCCCATT-3'

18S Rev 5'-CCATCCAATCGGTAGTAGCG-3'

Fig. S1

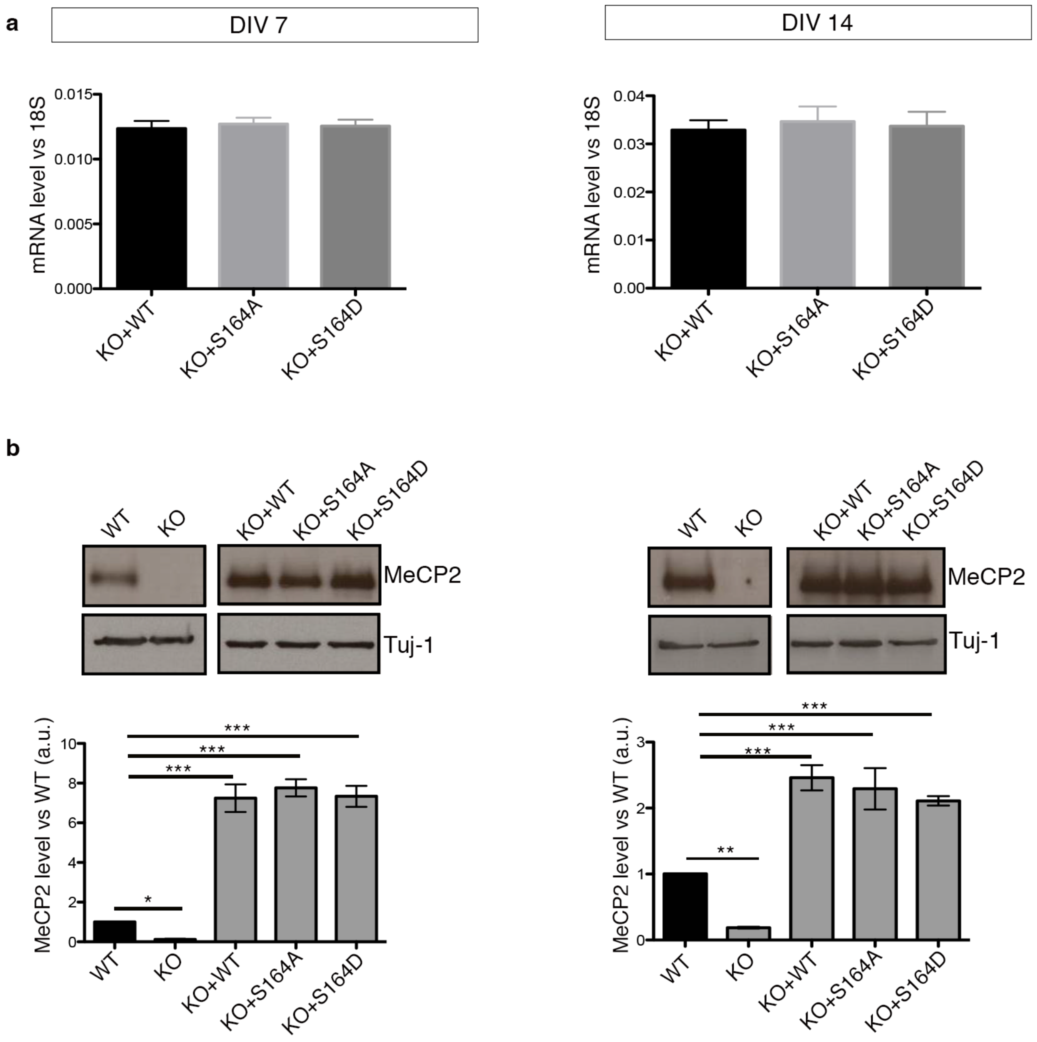

Fig. S1: For each experiment performed in Fig. 8, MeCP2 expression was carefully analyzed by extracting total proteins and RNAs. **(a)** Real time PCR analyses. The mRNA levels of human MeCP2 were normalized to 18S control (data are represented as mean ± SEM. *p=<0.05, **p=<0.01, ***p=<0.001, ****p=<0.0001, one way ANOVA n=4). **(b)** Western blot analysis of exogenously expressed MeCP2 derivatives at DIV7 and 14 (upper panels) using antibodies against total MeCP2. Tuj-1 was used as loading control. Lower panels show quantification of western blots (data are represented as

mean ± SEM, *p=<0.05, **p=<0.01, ***p=<0.001, ****p=<0.0001, one way ANOVA, *n*=3).

Fig. S2

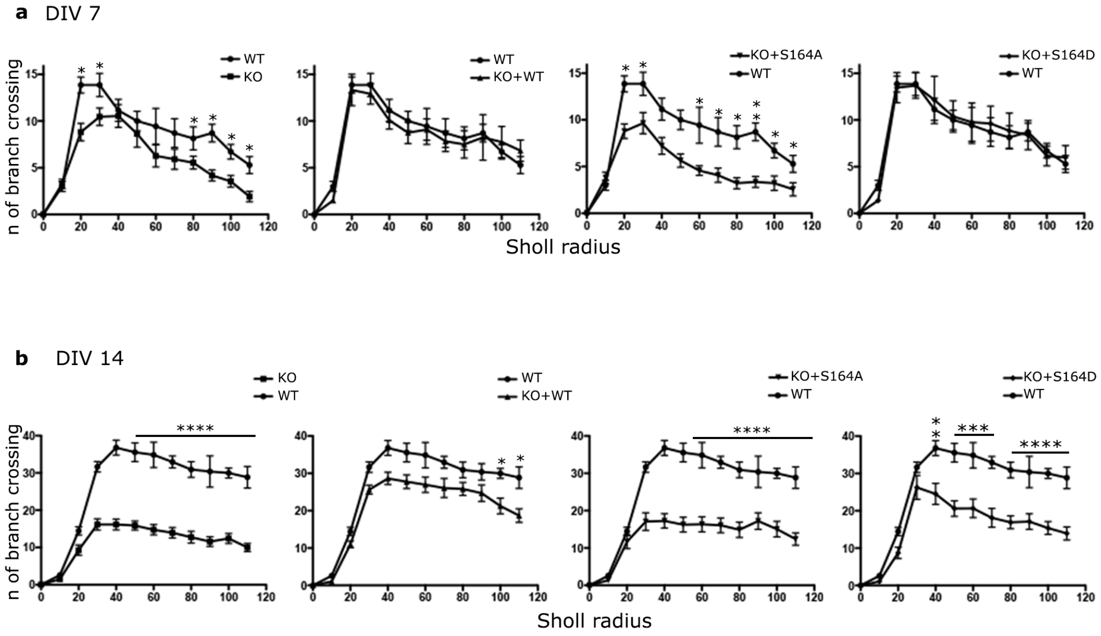

Supplementary Fig. S2: (a,b) Quantification of dendritic branch complexity by Sholl analysis at DIV7 (a) and 14 (b). All graphs report each condition compared to WT neurons for each time (data are represented as mean ± SEM, *p=<0.05, **p=<0.01, ***p=<0.001, ****p=<0.0001, one way ANOVA)(three animals per genotype; at least 20 neurons per condition).
