# Supplementary material for: Author Correction: Brain phosphorylation of MeCP2 at serine 164 is developmentally regulated and globally alters its chromatin association
Source: Sci Rep. 2021 May 26;11:11394. doi: 10.1038/s41598-021-90392-3 (PMC8154903; doi:10.1038/s41598-021-90392-3)
Supplement: Supplementary file 1 — Supplementary Information. [file 41598_2021_90392_MOESM1_ESM.pdf]

[illegible]

Brain phosphorylation of MeCP2 at serine 164 is developmentally regulated and globally alters its chromatin association

Gilda Stefanelli[1], Anna Gandaglia[1], Mario Costa[2], Manjinder S. Cheema[3], Daniele Dimarino[4], Isabella Barbiero[5], Charlotte Kilstrup-Nielsen[5], Juan Ausió[3] and Nicoletta Landsberger*,[1],[6]

[1]San Raffaele Rett Research Unit, San Raffaele Scientific Institute, Milan, Italy
[2]Institute of Neuroscience, National research council (CNR), Scuola Normale Superiore Pisa, Italy
[3]Department of Biochemistry and Microbiology, University of Victoria, Victor (BC), Canada
[4]Department of Informatics, Institute of Computational Science, Università della Svizzera Italiana, Lugano, Switzerland
[5]Department of Biotechnology and Life Sciences, University of Insubria, Bust Arsizio (VA), Italy
[6]Department of Medical Biotechnology and Translational Medicine, Universi of Milan, Milan, Italy

*corresponding author

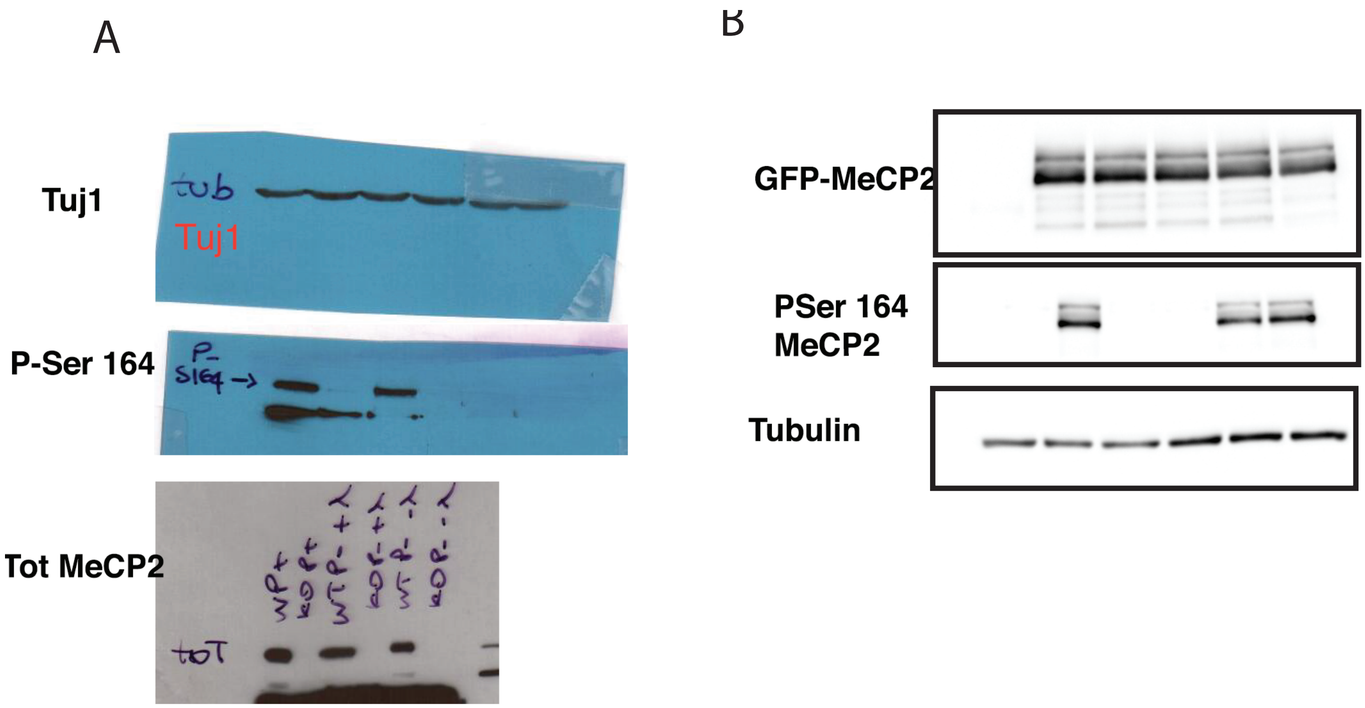

Fig S1: full blot of fig 1B (A) and Fig 1C (B)

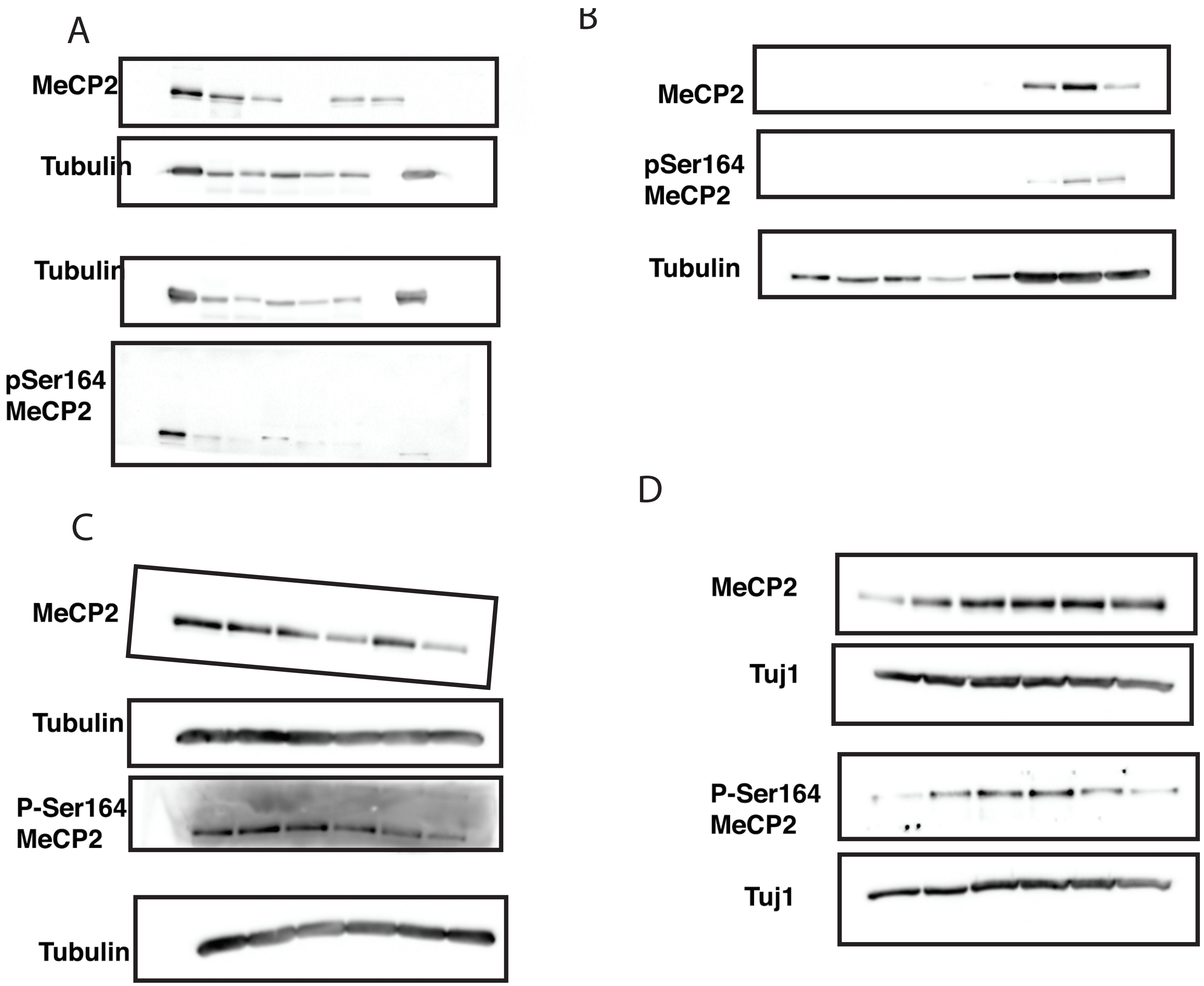

Fig S2: full blot of fig 2A(A), Fig 2B (B), 2C (C) and 2D (D)

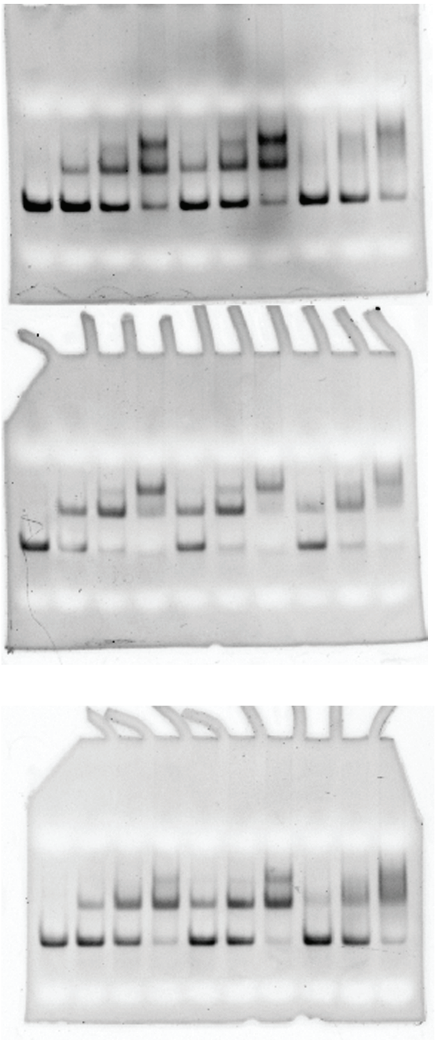

unmethylated

Methyl CG

Methyl CA

Fig S3: full gel of fig 5E

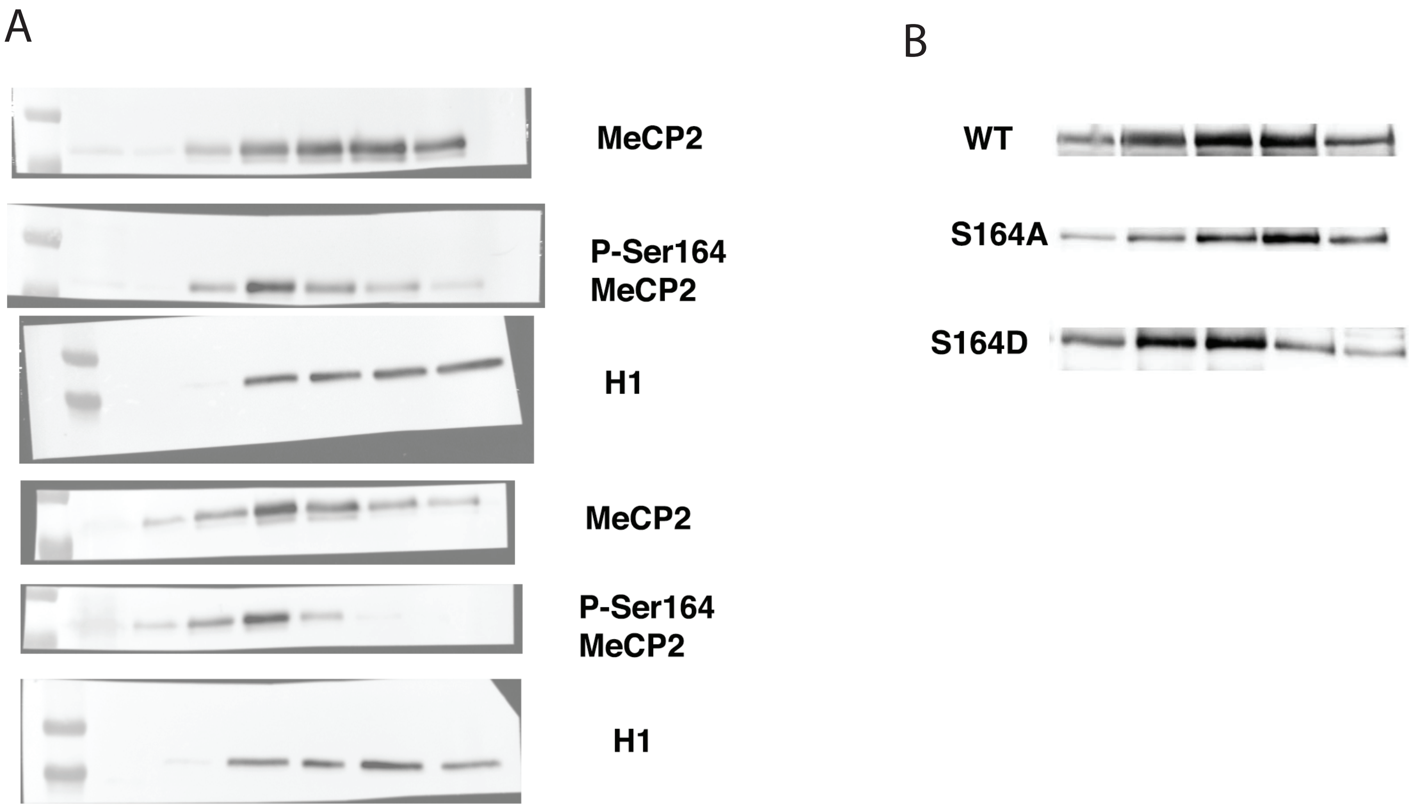

Fig S4: full blot of fig 6A (A) and 6B (B)

A

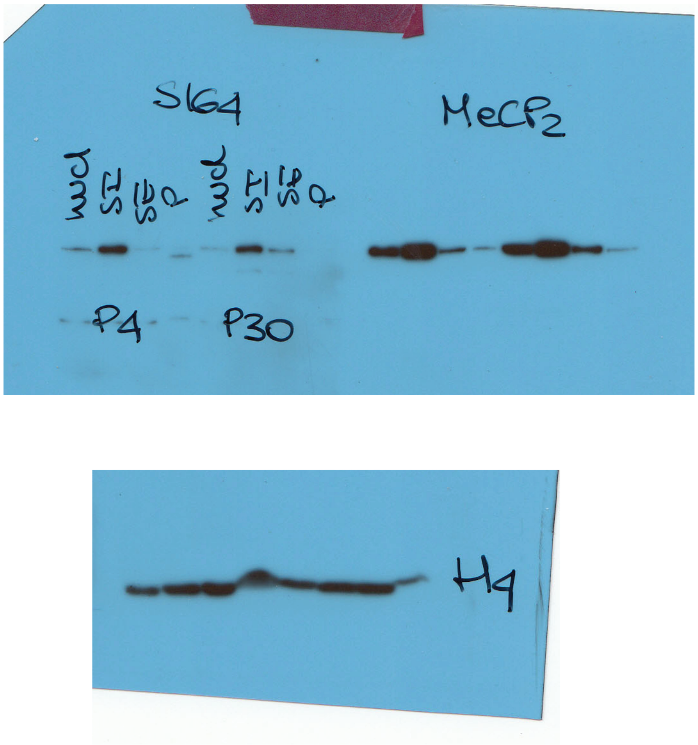
S164
MeCP2
P4
P30
H4

B

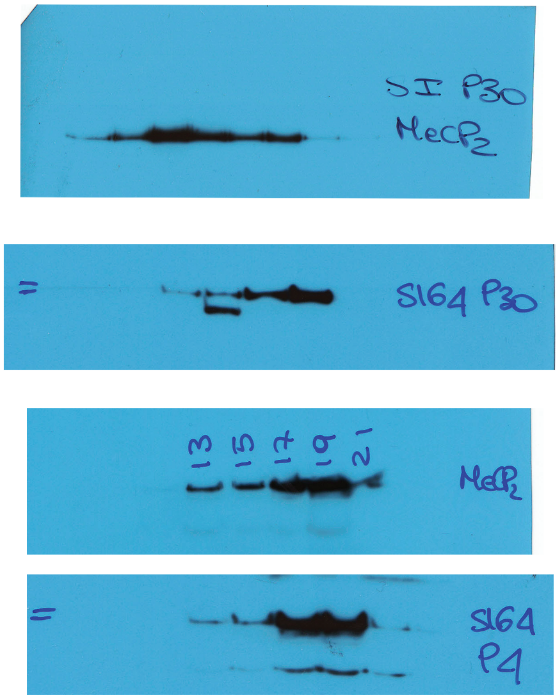
SI P30
MeCP2
S164 P30
13
15
17
19
21
MeCP2
S164
P4

C

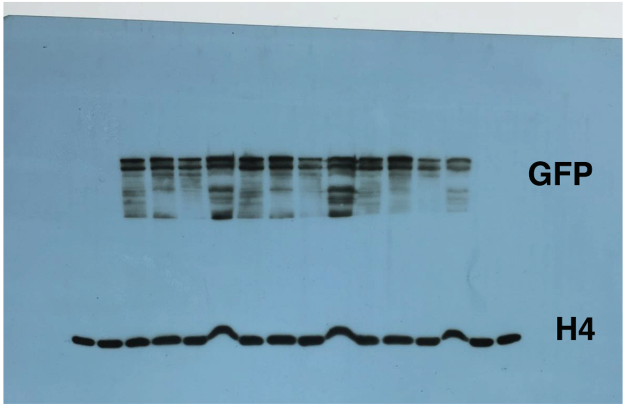
GFP
H4

D

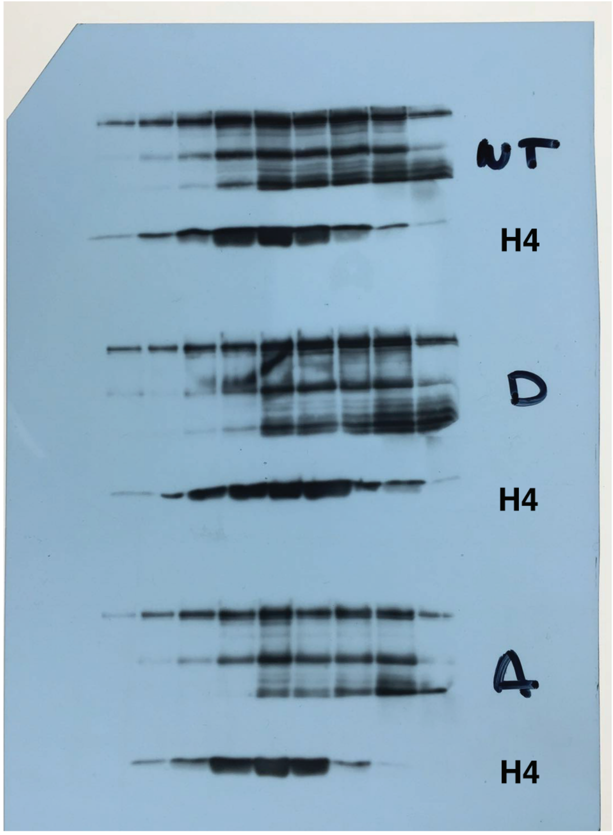
WT
H4
D
H4
A
H4
